# Prednisolone does not improve olfactory function after COVID-19: a randomized, double-blind, placebo-controlled trial

**DOI:** 10.1186/s12916-022-02625-5

**Published:** 2022-11-16

**Authors:** Emma J. A. Schepens, Esther E. Blijleven, Wilbert M. Boek, Sanne Boesveldt, Robert J. Stokroos, Inge Stegeman, Digna M. A. Kamalski

**Affiliations:** 1 Department of Otorhinolaryngology- Head and Neck Surgery, University Medical Center Utrecht, P.O. Box 85500, 3508 GA Utrecht, The Netherlands; 2 Brain Center, University Medical Center Utrecht, Utrecht, The Netherlands; 3 Department of Otorhinolaryngology- Head and Neck Surgery, Hospital Gelderse Vallei, Ede, The Netherlands; 4 Division of Human Nutrition and Health, Wageningen University, Wageningen, The Netherlands

**Keywords:** Smell, Olfactory function, COVID-19, Prednisolone

## Abstract

**Background:**

Prednisolone has been suggested as a treatment for olfactory disorders after COVID-19, but evidence is scarce. Hence, we aimed to determine the efficacy of a short oral prednisolone treatment on patients with persistent olfactory disorders after COVID-19.

**Methods:**

We performed a randomized, double-blind, placebo-controlled, single-centered trial in the Netherlands. Patients were included if they were > 18 years old and if they had persistent (> 4 weeks) olfactory disorders within 12 weeks after a confirmed COVID-19 test. The treatment group received oral prednisolone 40 mg once daily for 10 days and the placebo group received matching placebo. In addition, all patients performed olfactory training. The primary outcome was the objective olfactory function on Sniffin’ Sticks Test (SST) 12 weeks after the start of treatment, measured in Threshold-Discrimination-Identification (TDI) score. Secondary outcomes were objective gustatory function assessed by the Taste Strip Test (TST) and subjective self-reported outcomes on questionnaires about olfactory, gustatory and trigeminal function, quality of life, and nasal symptoms. The CONSORT 2010 guideline was performed**.**

**Results:**

Between November 2021 and February 2022, we included 115 eligible patients, randomly assigned to the treatment (*n* = 58) or placebo group (*n* = 57). No difference in olfactory function between groups was obtained after 12 weeks. Median TDI score on SST was 26.8 (IQR 23.6-29.3) in the placebo group and 28.8 (IQR 24.0-30.9) in the prednisolone group, with a median difference of - 1.5 (-3.0 to 0.25). There was similar improvement on olfactory function in both groups after 12 weeks. Furthermore, on secondary outcomes, we obtained no differences between groups.

**Conclusions:**

This trial shows that prednisolone does not improve olfactory function after COVID-19. Therefore, we recommend not prescribing prednisolone for patients with persistent olfactory disorders after COVID-19.

**Trial registration:**

This trial is registered on the ISRCTN registry with trial ID ISRCTN70794078.

## Background

Olfactory disorders are a common early feature in COVID-19 [1], occurring in about two of every three patients [2, 3]. Though most patients recover within 4 weeks [4], it is reported that up to ~ 46% of patients still have olfactory disorders after 6 months [5, 6] and 20–60% after a year [7, 8]. The prevalence of long-term olfactory disorders varies widely because of the different methods of assessing olfactory function and a lack of follow-up. Patients with persistent olfactory disorders can have increased depressive symptoms and nutritional issues, reducing patients’ quality of life [9].

At this time, the only therapeutic option for olfactory disorders in COVID-19 is olfactory training [10]. During olfactory training, a patient sniffs a set of known odors daily for a period of 6 months. Olfactory training may speed up and increase the extent of smell recovery [11, 12]; however, effects seem limited [13].

As the persistent loss of smell is thought to be caused by an inflammatory response [14], corticosteroids might be a treatment option. Some studies assessed corticosteroids in nasal spray, without beneficial effect [15–17]. Patients who were treated with a short oral prednisolone treatment experienced an improved sense of smell in two small studies [18–20]. However, these studies have a low level of evidence because of the limited number of cases (*n* = 9), short follow-up (4–10 weeks), and the non-blinded study designs. Due to the uncertainty of the evidence, there is still no consensus on treatment [20].

If treatment with prednisolone in combination with olfactory training more effectively improves olfactory function, a long-term disability may be prevented for more patients. Side effects of prednisolone, such as stomach irritations and nervousness/restlessness, need to be weighed against the potential benefit [21, 22].

Since many patients suffer from olfactory disorders after COVID-19, we need to ensure the effectiveness of this treatment. Therefore, we investigated the efficacy of a treatment in combination with olfactory training in a randomized, double-blind, placebo-controlled, single-centered trial.

## Methods

### Study design

**Fig. 1:**
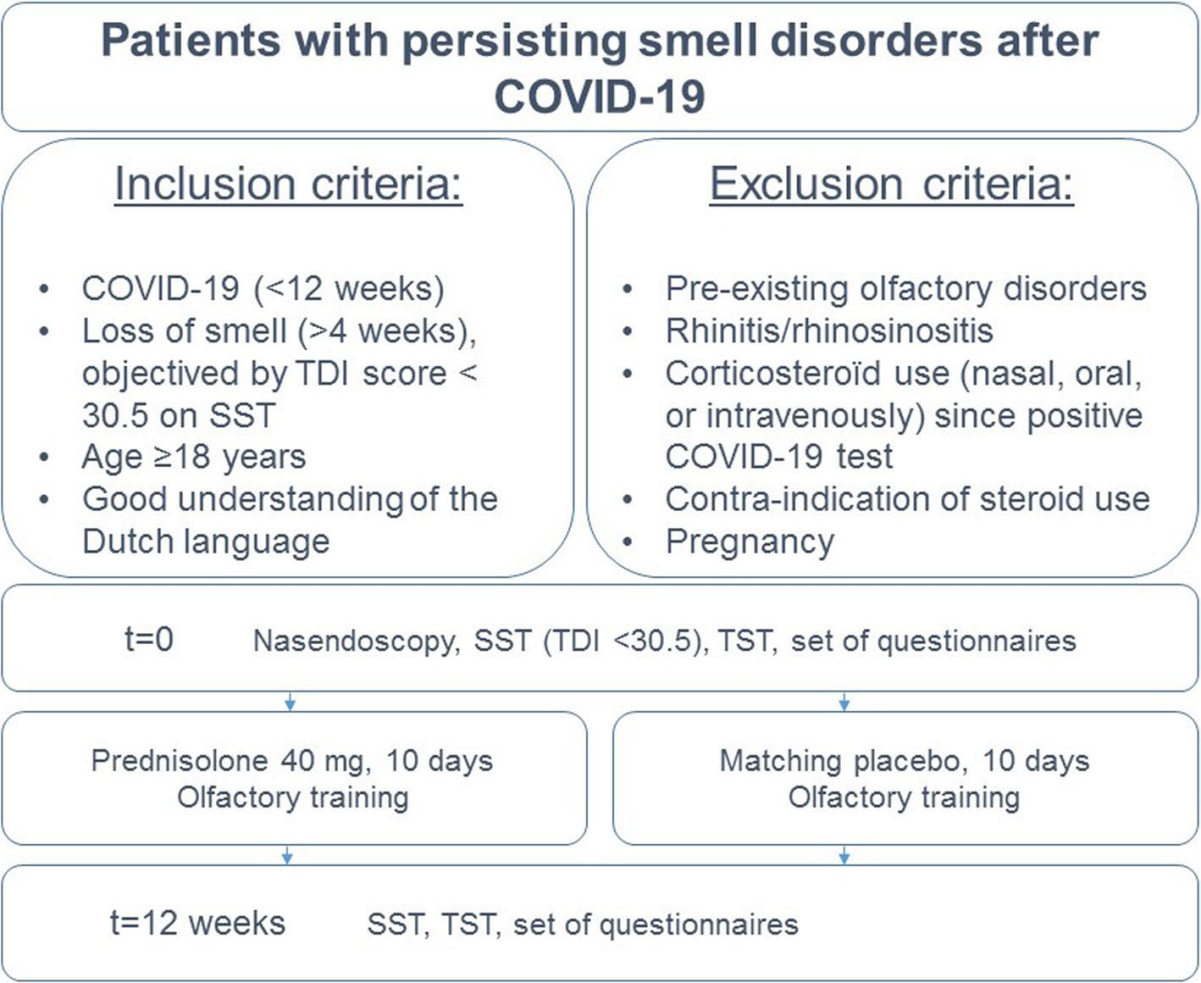
Study design. TDI score; Threshold-Discrimination-Identification score; SST; Sniffin’ Sticks Test; TST; Taste Strip Test

The corticosteroids for COVID-19 induced loss of Smell (COCOS) trial was a single-centered, randomized, double-blinded, placebo-controlled study in the Netherlands to determine the efficacy of a short prednisolone treatment on olfactory disorders after COVID-19. The trial consisted of a baseline visit at the outpatient Ear, Nose, and Throat (ENT) clinic and a second visit (follow-up) after 12 weeks (Fig. 1). The Institutional Review Board of the participating hospital approved the research protocol (protocol number: 21–635/G-D, October 2021). This study was conducted according to the principles of the Declaration of Helsinki (2013, Fortaleza). The CONSORT 2010 guideline was performed. The recruitment phase started November 2021 and ended February 2022. The trial ended May 2022.

### Participants

Participants were identified by the Dutch Patients Association for smell and taste disorders and via the Dutch media that had been approached by the participating hospital. Interested patients could contact the research team via our website during the screening period. Investigators contacted interested patients by telephone to check inclusion and exclusion criteria and medical status. Medical status consisted of medication use, medical history, date of confirmed positive COVID-19 test, and date of onset of the olfactory disorder. Patients were included if they were > 18 years old, if they had persistent (> 4 weeks) olfactory disorders within 12 weeks after COVID-19 diagnosis based on a positive test (PCR or antigen), and if they understood the Dutch language. Patients were excluded if they used oral anticoagulants without stomach protection or if they suffered from pre-existing olfactory disorders including chronic rhinitis or rhinosinusitis or diseases which contra-indicate the use of steroids (diabetes mellitus for which drugs are used, stomach ulcers/bleeding, psychoses or ongoing oncological disease). Women who were pregnant, or who intended to become pregnant, were excluded. Eligible patients were invited for a baseline visit at the outpatient ENT clinic at the participating hospital. At the baseline visit, patients could still be excluded if they had no objective hyposmia (reduced loss of smell) or anosmia (total loss of smell) confirmed with a Threshold-Discrimination-Identification (TDI) score > 30.5 on Sniffin’ Sticks Test (SST) or if they had other causes for olfactory disorders objectified by nasendoscopy.

### Procedures

We collected further baseline characteristics such as vaccination status and COVID-19 symptoms at first visit. Furthermore, we performed a nasendoscopy in order to eliminate other causes for olfactory disorders. Patients underwent objective smell and taste tests, and filled in three additional questionnaires. At the baseline visit, patients received their randomly allocated blinded study medication (40 mg prednisolone once daily for 10 days or matching placebo) and were instructed to start their 10 days of study medication the next morning. Researchers contacted patients by telephone ten days after the baseline visit to assess possible side-effects and treatment compliance. Patients in both groups performed 12 weeks olfactory training twice a day, coming to a total of 168 sessions. Patients crossed off a daily schedule allowing researchers to monitor olfactory training compliance. The follow-up visit was scheduled 12 weeks after the start of treatment. Outcome measurements were collected at the first visit (baseline) and second visit (follow-up) to compare outcomes (Fig. 1). All outcomes were registered in an electronic case report form (eCRF), the endorsed system Castor EDC.

### Randomization and blinding

Patients were randomly allocated to receive prednisolone or placebo. Half of the group was treated with capsules of 40 mg of prednisolone, once daily for 10 days. The other half received capsules of placebo, once daily for 10 days. The pharmacy that prepared prednisolone and placebo capsules made a block randomization sequence list, on which the patient subject number was linked to the study medication number. This pharmacy is a Dutch state-of-the-art good manufacturing practice compounding pharmacy independent from our department. To minimize seasonal effects between groups, randomization occurred in block sizes of four patients. The blinding of researchers, physicians, outcome assessors, and patients to the treatment allocation broke after all the analyses were finished.

### Outcome measures

The primary outcome was the objective difference between the two groups on the TDI score post-treatment at 12 weeks, measured with the Sniffin’ Sticks Test (SST, Burghart). The SST consists of three parts: a threshold test (score ranging 1–16), discrimination test (score ranging 0–16), and identification test (score ranging 0–16). The TDI score is the sum of these three tests and ranges from 1 to 48. The higher the score, the better the olfactory function. A score of ≤ 16.5 is considered as anosmia, a score of > 30.5 as normosmia, and scores between these values are considered as hyposmia. A difference of 5.5 on TDI score was determined as a clinically relevant difference for the primary outcome [23]. Secondary objective outcome was gustatory function measured by Taste Strip Test (TST, Burghart), assessing recognition thresholds and identification of the four basis tastes [24]. Total taste score range from 0 to 16 since scores for each taste range from 0 to 4. High scores indicate a better taste function. Clinical improvement was set at > 2 points [25].

Secondary subjective outcomes were olfactory, gustatory and trigeminal function, impact of smell/taste changes on quality of life, and nasal symptoms measured by subjective questionnaire outcomes. These contained the validated Sino-Nasal Outcome Test-22 questionnaire (SNOT-22) [26], self-reported smell, taste, trigeminal sensations questionnaire by medians of visual analog scale (VAS), ranging 0–10 [27], and the translated Olfactory Disorders Questionnaire (ODQ) [28, 29]. All outcomes where assessed during both first and second visit. Details of all outcomes, examinations, and questionnaires are reported in the protocol, Sect. 7.5 and 8.1.2 (appendix), http://dx.doi.org/10.1136/bmjopen-2021-060416.

### Statistical analyses

All statistical analyses were performed using the IBM SPSS Statistics 26.0.0.1 software and R statistical computing. We performed analysis on an intention-to-treat basis. Sample size was calculated based on means and standard deviations of an earlier pilot study [19]. With a power of 0.90, an alpha of 0.05, and a mean difference of 5.5 (SD 8.0) on SST-scores, the total sample size was 92. To correct for possible non-parametric testing, the sample size was increased with 15%. As the study is limited in time and effort for the patient, a maximum of 10% dropout was expected. This gives a total sample size of initial 116 patients, with 58 in every group. A test for normality was used to assess whether variables were normally distributed. Since all our outcomes were not-normally distributed, a Mann–Whitney *U* test was performed to determine statistical significant differences between the prednisolone and placebo group. The differences in continuous variables between the groups was calculated using Hodges-Lehmann estimation. Confidence intervals for differences between groups were reported.

### Patient and public involvement

The national patients association was involved in the conduct of the study, applying for the funding, and in recruiting patients. No patients were involved in the research questions or outcome measurements. We acknowledged and thanks the participants of our trial for their contribution. Patients who participate in this trial, who prefer, will be informed of the results.

## Results

### Patients

**Fig. 2:**
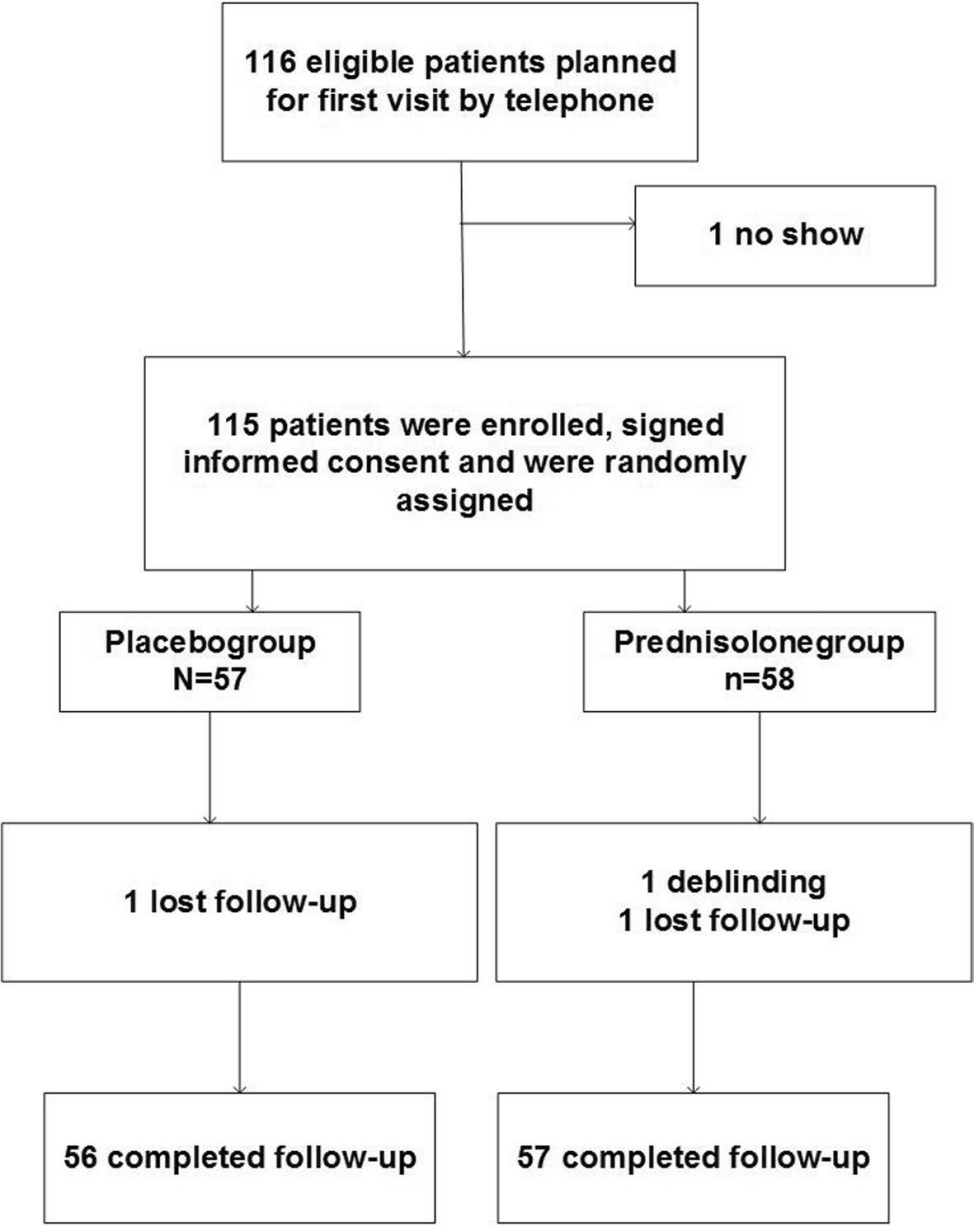
Participant flow-chart

In total, 115 eligible patients came for their first visit to the outpatient ENT department in the participating hospital. We initially planned 116 patients by telephone assessment, but there was one no show after the medication was already prescribed and collected from the pharmacy and could therefore not be reused. This led to 115 patients who were enrolled, who gave informed consent, and who were randomly assigned to the prednisolone group (*n* = 58) or placebo group (*n* = 57) (Fig. 2). There were no patients with a TDI score > 30.5 or abnormalities at nasendoscopy on first visit.

Trial participants were recruited during the fourth COVID-19 wave, presumably largely the Delta variant (July 2021 to January 2022) and were distributed from all over the Netherlands. Three patients had long-term COVID-19 related symptoms at the first visit such as fatigue, reduced cognitive function, and reduced physical condition which had all improved at second visit. The rest had experienced mild COVID-19 related symptoms such as cough, fever, or a cold during the infection or have had no complaints at all. No patients had been hospitalized.

### Baseline characteristics

Baseline characteristics are reported in Table 1. At baseline, there were no differences between age, gender, vaccination status, and time since confirmed positive COVID-19 test. Median age of all study patients was 49 years (IQR 41–57), with a minimum of 20 and a maximum of 78 years. Of these patients, 73 (63.5%) were female and 42 (36.5%) were male. In the placebo group were 50 patients (87.7%) vaccinated and in the prednisolone group 41 patients (70.7%). Median duration since confirmed COVID-19 test for all study patients was 56 days (IQR 44–69), with 53 days (IQR 43.5–67.0) in the placebo group and 59.5 days (IQR 46.5–73.0) in the prednisolone group. Median objective TDI score in the placebo group was 20.5 (IQR 17.5–24.3) and 22.9 (IQR 19.9–25.1) in the prednisolone group. Median self-reported smell function on VAS-score was 1.1 (IQR 0.3–3.1) in the placebo group and 1.4 (IQR 0.5–2.8) in the prednisolone group. Both objective and subjective gustatory function did not differ at baseline between the two groups. Scores on TST were equal in both groups with a median of 10 (IQR 7–12), and the median self-reported taste function on VAS-score was 3.4 (1.2–5.8) in the placebo group and 3.7 (IQR 1.0–5.8) in the prednisolone group.

**Table 1:** Baseline characteristics, intention to treat population

	**Placebo group (*n* = 57)**	**Prednisolone group (*n* = 58)**
Age, years	46 (39.5–55.0)	51 (42.5–59.3)
Sex		
Female	39 (68.4%)	34 (58.6%)
Male	18 (31.6%)	24 (41.4%)
Vaccinated	50.0 (87.7%)	41 (70.7%)
Time since positive COVID-19 test, days	53.0 (43.5–67.0)	59.5 (46.5–73.0)
Sino-nasal Outcome Test (SNOT-22)	23.0 (14.5–37.0)	20.5 (13.5–44.0)
Sniffin’ Sticks Test (SST)		
Threshold-Discrimination-Identification (TDI) score	20.5 (17.5–24.3)	22.9 (19.9–25.1)
Threshold	1.3 (1.0–3.4)	1.5 (1.0–3.8)
Discrimination	9 (7–11)	10 (8–11)
Identification	9 (7–11)	10 (9–12)
Taste Strip Test (TST)		
Total score	10 (7–12)	10 (7–12)
Sweet	3 (2–4)	4 (2.8–4)
Sour	2 (1–3)	2 (1–3)
Salty	2 (1–3)	3 (2–3)
Bitter	3 (1–3)	2 (1–3)
Olfactory Disorders Questionnaire (ODQ)		
Total score	0.5 (0.4–0.6)	0.5 (0.4–0.5)
Self–reported visual analog scale (VAS)		
Sense of smell	1.1 (0.3–3.1)	1.4 (0.5–2.8)
Sense of taste	3.4 (1.2–5.8)	3.7 (1.0–5.8)
Trigeminal sensations	3.8 (2.1–5.8)	5.2 (2.6– 6.8)
Olfactory training compliance, frequency	136 (101–150)	123 (83–152)

Moreover, we obtained no differences between group on quality of life, nasal symptoms, or self-reported trigeminal function (Table 1).

Data are presented as median (IQR) or *n* (%), except where otherwise stated. Outcome ranges were as follows: SNOT-22 0–50; TDI 1–48; T 1–16; D; 0–16; I 0–16; TST 0–16; Sweet, Sour, Salty, Bitter 0–4; ODQ 0.13–1.0;VAS 0–10; Frequency Olfactory training 0–168.

### Follow-up

After 12 weeks, all patients were eligible for follow-up since analysis was performed on the intention-to-treat base. Two patients were lost to follow-up (Fig. 2). One patient in the placebo group had to receive a prednisolone treatment for her asthma (7 days of 20 mg prednisolone once daily), between first and second visit, but after 10 days of study treatment.

### Outcomes

Primary and secondary outcomes are presented in Table 2. At 12 weeks of follow-up, patients treated with prednisolone showed no better olfactory function than patients treated with placebo. Median TDI score was 26.8 (IQR 23.6- 29.3) in the placebo group and 28.8 (IQR 24.0- 30.9) in the prednisolone group, with a median difference of - 1.5 (-3.0 to 0.25). There was similar improvement on olfactory function in both groups after 12 weeks. Separate TDI scores did not show any significant or clinically relevant difference (Table 2). Self-reported smell function on VAS-score was 3.2 (IQR 1.8- 6.5) in the placebo group and 3.6 (IQR 1.0- 5.8) in the prednisolone group with a median difference of 0.3 (95% CI -0.9 to -1.3, *p* = 0.53). Additionally, no effect was obtained on objective gustatory function in the prednisolone group compared to the placebo group. Both groups showed a median of 11 on TST (IQR 9–13, *p* = 0.50). Self-reported taste function on VAS-score was 5.6 (IQR 2.3- 7.6) in the placebo group and 5.0 (IQR 2.0- 7.8) in the prednisolone group with a median difference of 0.1 (95% CI -1.0 to 1.3, *p* = 0.80). Moreover, no differences between groups were obtained in quality of life, nasal symptoms, or self-reported trigeminal function on questionnaires (Table 2).

**Table 2:** Primary and secondary outcomes at 12 weeks

	**Placebo group (*n* = 56)**	**Prednisolone group (*n* = 57)**	**Difference (95% CI)**	***P* value**
Sniffin’ Sticks Test (SST)				
Threshold-Discrimination-Identification (TDI) score	26.8 (23.6–29.3)	28.8 (24–30.9)	- 1.5 (-3.0 to 0.25)	*p* = 0.10
Threshold	4.3 (3.3–5.4)	4.5 (3.1–6.4)	-0.25 (-1.0 to 0.5)	*p* = 0.47
Discrimination	11 (10–12)	12 (10.5–13)	-1.00 (-1.00 to 0.00)	*p* = 0.12
Identification	11.5 (10–12)	11 (10–13)	0.00 (-1.00 to 1.00)	*p* = 0.45
Taste Strip Test (TST)				
Total score	11 (9.3–13)	11 (9–13)	0.00 (-1.00 to 1.00)	*p* = 0.50
Sweet	4 (3–4)	4 (3–4)	0.00 (0.00 to 0.00)	*p* = 0.66
Sour	2 (2–3)	2 (2–3)	0.00 (0.00 to 0.00)	*p* = 0.84
Salty	3 (2–4)	3 (2–4)	0.00 (0.00 to 1.00)	*p* = 0.31
Bitter	3 (2–4)	3 (2–3.5)	0.00 (0.00 to 1.00)	*p* = 0.47
Sino-nasal Outcome Test (SNOT-22)	16 (10–26)	19 (10–32)	-1.00 (-7.0 to 4.0)	*p* = 0.69
Olfactory Disorders Questionnaire (ODQ)				
Total score	0.4 (0.3–0.6)	0.4 (0.3–0.5)	0.00 (-0.06 to 0.06)	*p* = 0.89
Self-reported visual analog scale (VAS)				
Sense of smell	3.2 (1.8–6.5)	3.6 (1.0–5.8)	0.3 (-0.9 to 1.3)	*p* = 0.53
Sense of taste	5.6 (2.3–7.6)	5.0 (2.0–7.8)	0.1 (-1.00 to 1.3)	*p* = 0.80
Trigeminal sensations	5.1 (2.9–7.4)	5.3 (2.4–7.9)	-0.2 (-1.3 to 1.00)	*p* = 0.76

The daily olfactory training schedule was not obtained in 2 of 113 patients. There were no major differences between compliance of olfactory training between the groups. Compliance of olfactory training is expressed in frequencies (Table 1).

Data are presented as median (IQR) or *n* (%), except where otherwise stated. Differences are expressed as rate differences between groups or Hodges-Lehmann estimator and 95% confidence interval. Outcome ranges were as follows: SNOT-22 0–50; TDI 1–48; T 1–16; D; 0–16; I 0–16; TST 0–16; Sweet, Sour, Salty, Bitter 0–4; ODQ 0.13–1.0;VAS 0–10; Frequency Olfactory training 0–168.

### Harms

We reported three adverse events in the prednisolone group in Table 3. Adverse events contained severe side-effects for which intervention, discontinuation, or deblinding of treatment was needed. No serious adverse events occurred. For one patient in the prednisolone group, we requested to break the blinding of the study medication due to psychological disorders and sleeplessness after full treatment compliance. This was the only patient with a deblinding before the end of the study. Three patients stopped the treatment because of side-effects after a minimum of six days, of which two patients were allocated in the placebo group and one in the prednisolone group. The rest of the patients in both groups complied with their treatment.

**Table 3:** Adverse events

Adverse events	*N* = 3
Sleeplessness with psychological disorders after 10 days of treatment for which deblinding of treatment was broken	1
Restlessness for which patient stopped treatment after 8 days	1
Stomach irritation for which omeprazole was needed, full treatment compliance	1

We reported 14 patients with mild side-effects, of which 9 (15.5%) in the prednisolone group (*n* = 58) and five (8.8%) in the placebo group (*n* = 57). The most reported side-effects were nervousness/restlessness and stomach irritation. All side-effects were mild, common, and lasted a short time or stopped immediately after finishing the ten days of treatment.

## Discussion

This randomized double-blind, placebo-controlled trial for patients with persistent olfactory disorders after COVID-19 showed that patients who received a short prednisolone treatment had no better olfactory function than patients who received placebo treatment.

Two previous studies did show a possible better olfactory function in patients who received a short prednisolone treatment [18, 19]. This study failed to support that claim. The reason for the discrepancy in outcomes is most likely due to the biases included in these previous studies. Only nine patients were treated in each study on their own request. The studies were not blinded or randomized. Even though both studies used an objective outcome measure of smell, the investigator taking the test might have influenced the outcome. In our study design, we eliminated these biases and ensured sufficient power. There are however limitations in our trial we have to take in consideration. In our study, we treated patients with 10 days of 40 mg prednisolone, starting at least 4 weeks (median ~ 59.5 days) after the initial infection. Prednisolone dosage and timing could have influenced outcome. With the limited available evidence, we choose to use a comparable dosage schedule as used in the previous studies. Higher dosage might have increased effectiveness, but also would have increased side effects, both in number and severity. The short prednisolone treatment schedule is well known in otorhinolaryngology practice. The same schedules are used in sensorineural hearing loss and Bell’s palsy. However, in these diseases, prednisolone treatment starts preferably within 72 h after the start of symptoms [21, 22]. Nevertheless, we started treatment after 4 weeks in this trial. Firstly, because most patients regain spontaneous normal smell and taste function within this 4-week period, treating them in that phase could risk overtreatment. Secondly, the immune system against COVID-19 can be inhibited by prednisolone which can lead to a prolonged infection.

Due to the timing of this study, it is likely that mainly patients with the COVID-19 Delta variant are included, although we did not test for this specifically. Up to now, it is unknown how different COVID variants influence outcome in olfactory function. The Omicron variant though has proven to have less negative effect on smell and taste than the Delta variant [30]. Possibly and hopefully, fewer Omicron patients will face long-term disability in olfactory disorders, compared to Delta patients. We presume that it is unlikely that the COVID variants influenced the outcome of this study.

The only current treatment option for persistent olfactory disorders after COVID-19 is olfactory training. In this study, both groups improved substantially on their olfactory function on the second visit. This suggests that even after a longer period of time, smell will continue to improve. Therefore, we intend to retest our study population 1 year after the initial infection, to gain better insight into the course of the olfactory function. Furthermore, if we gain a better understanding of the pathophysiological mechanisms underlying olfactory disorders, we may be able to develop new treatments.

## Conclusions

Our randomized, double-blind, placebo-controlled trial showed no beneficial effect of a prednisolone treatment (40 mg daily, for 10 days) over placebo treatment in patients with persisting olfactory disorders (> 4 weeks) after COVID-19 (< 12 weeks). Therefore, we recommend not to prescribe prednisolone for patients with olfactory disorders after COVID-19. However, we have to take in consideration that our trial assessed outcomes on a specific population, treatment dosage, and time. As variants changes, as countries may have different COVID-19 treatment protocols, results may vary. Other studies focusing on different treatment schedules, severity of illness, and COVID-19 variants could help to confirm our findings.

## Data Availability

Participant data are available from the corresponding author under reasonable request.

## Abbreviations

COVID-19: Coronavirus disease 2019
COCOS: Corticosteroids for COVID-19 induced smell loss
ENT: Ear, Nose and Throat
CONSORT: Consolidated Standards of Reporting Trials
PCR: Polymerase chain reaction
TDI: Threshold-Discrimination-identification
SST: Sniffin’ Sticks Test
eCRF: Electronic case report form
EDC: Electronic data capture
TST: Taste Strip Test
SNOT-22: Sino-Nasal Outcome Test -22
VAS: Visual analog scale
ODQ: Olfactory Disorders Questionnaire
IBM SPSS: IBM software for Statistical Package for the Social Sciences
Sd: Standard deviation
IQR: Interquartile range
CI: Confidence interval
