# Correction: Prednisolone does not improve olfactory function after COVID-19: a randomized, double-blind, placebo-controlled trial

**DOI:** 10.1186/s12916-023-02786-x

**Published:** 2023-02-16

**Authors:** Emma J. A. Schepens, Esther E. Blijleven, Wilbert M. Boek, Sanne Boesveldt, Robert J. Stokroos, Inge Stegeman, Digna M. A. Kamalski

**Affiliations:** 1 Department of Otorhinolaryngology- Head and Neck Surgery, University Medical Center Utrecht, P.O. Box 85500, 3508 GA Utrecht, The Netherlands; 2 Brain Center, University Medical Center Utrecht, Utrecht, The Netherlands; 3 Department of Otorhinolaryngology- Head and Neck Surgery, Hospital Gelderse Vallei, Ede, The Netherlands; 4 Division of Human Nutrition and Health, Wageningen University, Wageningen, The Netherlands

**Correction: BMC Medicine 20, 445 (2022)**

**https://doi.org/10.1186/s12916-022-02625-5**

The original article [1] contained the following errors with their respective amendments:

1) Abstract, lines 48 and 49:

“Median TDI score on SST was 26.8 (IQR 23.6-29.3) in the placebo group and 28.8 (IQR 24.0-30.9) in the prednisolone group, with a median difference of 2.0 (95% CI 0.75 to 1.5).”

to

“Median TDI score on SST was 26.8 (IQR 23.6-29.3) in the placebo group and 28.8 (IQR 24.0-30.9) in the prednisolone group, with a median difference of - 1.5 (-3.0 to 0.25).”

2) Outcome, line 233-235:

“Median TDI score was 26.8 (IQR 23.6- 29.3) in the placebo group and 28.8 (IQR 24.0- 30.9) in the prednisolone group, with a median difference of 2.0 (95% CI 0.75 to 1.5, p = 0.10).”

to

“Median TDI score was 26.8 (IQR 23.6- 29.3) in the placebo group and 28.8 (IQR 24.0- 30.9) in the prednisolone group, with a median difference of - 1.5 (-3.0 to 0.25).”

3) Line 237-238:

"Self-reported smell function on VAS-score was 3.2 (IQR 1.8- 6.5) in the placebo group and 3.6 (IQR 1.0- 5.8) in the prednisolone group with a median difference of 0.45 (95% CI 1.1 to -1.2, p = 0.53)."

to

"Self-reported smell function on VAS-score was 3.2 (IQR 1.8- 6.5) in the placebo group and 3.6 (IQR 1.0- 5.8) in the prednisolone group with a median difference of 0.3 (95% CI -0.9 to -1.3, p = 0.53)."

4) Line 240-242:

“Self-reported taste function on VAS-score was 5.6 (IQR 2.3- 7.6) in the placebo group and 5.0 (IQR 2.0- 7.8) in the prednisolone group with a median difference of 0.25 (95% CI 0.1 to -0.2, p = 0.80).”

to

“Self-reported taste function on VAS-score was 5.6 (IQR 2.3- 7.6) in the placebo group and 5.0 (IQR 2.0- 7.8) in the prednisolone group with a median difference of 0.1 (95% CI -1.0 to 1.3, p = 0.80).”
